# Caries risk assessment in young adults: a 3 year validation of the Cariogram model

**DOI:** 10.1186/1472-6831-15-17

**Published:** 2015-01-27

**Authors:** Gunnel Hänsel Petersson, Svante Twetman

**Affiliations:** Department of Cariology, Faculty of Odontology, Malmö University, SE-205 06 Malmö, Sweden; Department of Cariology, Endodontics, Pediatric Dentistry and Clinical Genetics, Faculty of Health and Medical Sciences, University of Copenhagen, Copenhagen, Denmark; Maxillofacial Unit, Halland Hospital, Halmstad, Sweden

**Keywords:** Lactobacilli, Mutans streptococci, Risk factors, Saliva

## Abstract

**Background:**

To validate baseline caries risk classifications according to the Cariogram model with the actual caries development over a 3-year period in a group of young adults living in Sweden.

**Methods:**

The study group consisted of 1,295 19-year-old patients that completed a comprehensive clinical baseline examination, including radiographs and salivary tests. An individual caries risk profile was computed and the patient was placed in one of five risk categories. After 3 years, 982 patients (75.8%) were re-examined and caries increment for each patient was calculated. The outcome was expressed as sensitivity, specificity and predictive values and compared with a risk assessment scheme used in Public Dental Service.

**Results:**

The drop-outs displayed more risk factors and a significantly higher caries burden at baseline compared with those that remained in the project (p < 0.05). There was a strong association between the Cariogram risk categories and the 3-year caries increment on cavity level but the predictive values were modest. The high or very high caries risk categories yielded high specificities (>90%) but poor sensitivities. The low risk groups displayed higher sensitivities on expense of impaired specificities. No combinations proved clinically useful values according to Yuoden’s index.

**Conclusions:**

Within the limitations of the present study, the computer-based Cariogram did not perform better than a caries risk assessment scheme based on past caries experience and caries progression, over a 3-year period in young adults.

## Background

Caries risk assessment (CRA) is the clinical process of establishing the probability for an individual patient to develop caries lesions in the near future and thereby an essential component in the decision-making process for adequate prevention and management of dental caries [1–3]. In comprehensive clinical practice, risk factors based on general health, diet, oral hygiene, fluoride exposure and past caries experience are often subjectively and intuitively merged into one of several risk categories [4, 5], albeit the quality of evidence for this process is limited [6]. Cariogram is an algorithm based software based on nine different caries related risk factors and intended to aid clinicians in performing more objective and consistent risk assessments [7]. The performance of the program has been validated in preschool children [8], schoolchildren [9–12] and elderly [13]. To our knowledge, the accuracy of Cariogram in young adults has only been described in one previous study with a limited sample size [14]. The aim of the present study was therefore to validate baseline caries risk classifications according to Cariogram with the actual caries development over a 3-year period in a group of young adults living in Sweden. A second aim was to compare the outcome with a caries risk assessment scheme used within Public Dental clinics (PDC) within the region of Skåne as previously described [15].

## Methods

### Study group

**Figure 1:**
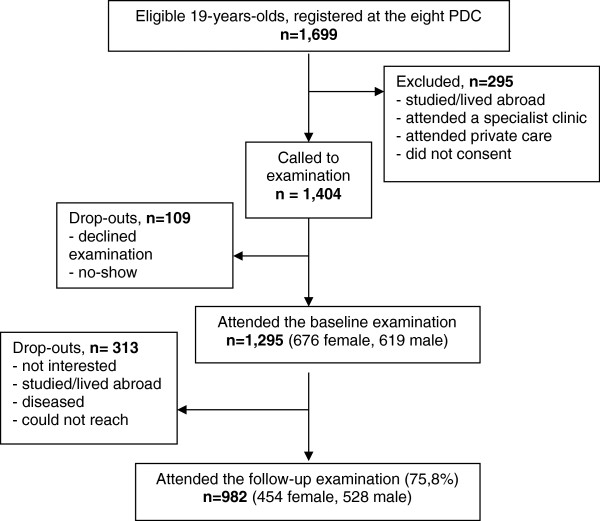
**Flow-chart indicating attrition and drop-outs.**

An invitation to take part in a prospective study was sent to all public dental clinics in the Skåne region, located in southern Sweden. From the positive responses, eight clinics were selected to represent various geographic and socioeconomic areas of the region. All 19-year-olds registered at the selected clinics were invited (n = 1,699) and 1,295 subjects were enrolled after verbal and written information. Further details on the selection of the baseline material as well as its characteristics have been published before [15]. A thorough baseline examination including radiographs and saliva sampling was conducted by the patients’ ordinary dental team, as detailed below. After 3 years, 982 patients (75.8%) were re-examined by the baseline team when possible. A flow-chart with the main reasons for drop-out is shown in Figure 1. All the patients were residents in areas with low natural fluoride content in the drinking water supply but the vast majority reported use of fluoridated dentifrice on regular basis. The study design was approved by the Ethical Committee, Lund University, Sweden.

### Clinical examination

Each selected clinic was visited by the principal investigator (GHP) and the dental personnel were informed on the aim and lay-out of the study. The clinical visual-tactile examination, including bitewing radiographs, was carried out by the regular dentist or dental hygienist under optimal light and cleaned, air-dried teeth. Caries prevalence and experience was registered at manifest dentin level according to the WHO-criteria [16] and expressed as DFT/DFS. Information concerning general health and medication, diet and oral hygiene habits including tooth brushing frequency and the use of fluoride was collected through a structured questionnaire. Paraffin-stimulated whole saliva was collected for 5 minutes and the secretion rate was expressed as ml/min. Salivary mutans streptococci, lactobacilli and buffer capacity were estimated by selective chair-side kits (Dentocult® SM-Strip mutans, Dentocult® LB and Dentobuff® Strip; Orion Diagnostica, Espoo, Finland) according to the manual of the manufacturer.

### Caries risk assessment with Cariogram

Data for the computerised caries risk assessment were entered into the Cariogram to obtain an individual caries risk profile as previously described [6, 9]. The following five Cariogram categories were used: “very low risk” = 81-100% chance to avoid caries; “low risk” = 61-80% chance to avoid caries; “moderate risk” = 41-60% chance to avoid caries; “high risk” = 21-40% chance to avoid caries; and “very high risk” = 0-20% chance to avoid caries. The calculated Cariogram risk category was not unveiled to neither to the patient nor the patient’s ordinary dental team. All decisions on preventive and restorative dental care were solely the responsibility of the patient’s regular public dental team during the entire study period.

### Endpoints

The endpoints were the number of new carious lesions in each risk category over the three-year study period and the calculated caries-predictive values for the various risk groups. Caries increment was determined by comparing the caries status for each patient registered at the follow-up with baseline. Thus, the caries increment was computed by counting the number of surfaces that changed from sound to decayed or filled over the study period. Possible caries reversals were not considered.

### Statistical methods

All data were processed with the IBM-SPSS software (version 19.0, Chicago, IL, USA). Descriptive statistics and correlations were applied. Comparisons and associations concerning caries data were conducted with one-way ANOVA and chi-square tests. Sensitivity, specificity and predictive values were calculated from two-by-two tables. *P*-values less than 0.05 were considered statistically significant.

## Results

The mean caries frequency and the percentage distribution of the Cariogram risk categories at baseline for all children, the dropouts and those that were re-examined after 3 years is shown in Table 1. The drop-outs had significantly higher mean values of DFT and DFS compared with those that remained in the project (p < 0.05). Likewise, more dropouts (24%) were considered “high risk” and “very high risk” according to the Cariogram categories compared with 15% in the final material. The 3-year caries increment in the five Cariogram categories is presented in Table 2. The positive relationship was statistically significant; the patients in the higher risk categories developed more new caries lesions than those assessed with lower caries risk (p < 0.05). The difference between the two highest risk groups (“high risk” and “very high risk”) was however not statistically significant. The distribution of patients with new caries lesions vs. no new caries lesions in relation to the baseline Cariogram risk category is shown in Table 3 and the sensitivity, specificity and predictive values are displayed in Table 4. The specificity was high (>90%) for those assessed with 0-40% chance to avoid caries but the sensitivity was poor. On the other hand, when the “low caries risk” category was used as a cut-off level, a high sensitivity and high negative predictive value was obtained. No combinations reached however high and clinically useful values according to Youden’s index.

**Table 1:** **Baseline caries frequency (mean, SD) and percentage distribution of Cariogram risk categories in all patients, the drop outs and those that remained after 3 years (follow-up)**

Variable	All patients	Drop outs	Follow-up
	n = 1,295	n = 313	n = 982
**DFT**	3.4 (3.3)	4.4^a^ (3.7)	3.1 (3.1)
**DFS**	4.9 (5.6)	6.8^a^ (6.9)	4.3 (5.0)
**Cariogram risk category (%)**			
81-100 (very low risk)	23.3	17.0	25.6
61-80	32.7	27.7	34.1
41-60	26.5	30.9	25.3
20-40	9.0	13.2	7.5
0-20 (very high risk)	8.4	11.2	7.5

^a^Significantly different from follow-up group (p < 0.05).

**Table 2:** **Mean caries increments (Δ) over 3 years expressed as mean DFT and DFS in the various Cariogram risk categories**

Risk group at baseline	n	ΔDFT (SD)	ΔDFS (SD)	ΔDFS = 0 (%)
81–100 (very low risk)	251	0.24 (0.58)	0.29 (0.89)	85.3
61–80	335	0.53 (1.07)	0.85 (1.91)	71.3
41–60	248	0.82 (1.18)	1.59 (2.55)	50.4
21–40	74	0.84 (0.95)	1.70 (1.76)	36.5
0–20 (very high risk)	74	1.00 (1.40)	1.99 (3.00)	44.6
ANOVA/Chi-square	p < 0.001	p < 0.001	p < 0.001	p < 0.001

Values in parenthesis denote the standard deviation.

**Table 3:** **Distribution of patient’s with new and no new caries lesions over 3 years in relation to the Cariogram risk category at baseline**

Baseline risk category	ΔDFS > 0	ΔDFS = 0	Sum
81-100 (very low risk)	37 (14.7)	214 (85.3)	251
61-80	96 (28.7)	239 (71.3)	335
41-60	123 (49.6)	125 (50.4)	248
21-40	47 (63.5)	27 (36.5)	74
0-20 (very high risk)	41 (55.4)	33 (44.6)	74
Sum	344 (35.0)	638 (65.0)	982

The values in the table denote number of patients and per cent within each risk category.

**Table 4:** **Sensitivity, specificity and predictive values for new caries lesions (ΔDFS > 0) over 3 years**

Cariogram cut-off, %	TP + TN^a^	Sensitivity	Specificity	PPV^b^	NPV^b^	Youden’s index^c^
80	53.1	89.2	33.5	42.0	85.3	0.23
60	65.8	61.3	71.0	53.3	77.3	0.32
40	67.8	25.6	90.6	59.5	69.3	0.16
20	65.8	11.9	94.8	55.4	66.6	0.07

^a^Proportion of true positive (TP) and true negative (TN) tests.

^b^PPV = positive predictive value; NPV = negative predictive value.

^c^J = sensitivity + specificity −1.

## Discussion

The present prospective study evaluated a computer-based caries risk assessment program in an age group seldom studied. We have earlier described the risk assessment process performed according to guidelines of the public dental clinics (PDC) in the same material [17] and the simple conclusion is that the Cariogram did not improve the accuracy of the assessments in this age group. The PDC risk assessment relied basically on past caries experience and progression of proximal enamel lesion. This concept was supported by recent systematic reviews that have suggested baseline caries prevalence as the most accurate single predictor of caries risk in all age groups [6] and unveiled limited or weak evidence for existing caries risk systems [18]. In contrast, Gao and co-workers [19] supported algorithm-based models before reasoning-based programs but their findings were based on a preschool material. The fact that the specificity was higher than the sensitivity at most cut-off levels indicated that selecting out those with low caries risk might be a more fruitful strategy than finding individuals with high risk. Notable, the Cariogram model has performed better in some previous reports, and especially in schoolchildren [10, 12]. In the study of Celik and coworkers [14], 100 young adults were followed for two years but no predictive values were reported. Nevertheless, the findings of the present study did not motivate the time and costs of saliva sampling and microbial cultivation if only the predictive values were considered. Still, the Cariogram may be helpful in patient motivation and communication of the preventive message.

Before disqualifying the Cariogram however, there are three issues that need to be addressed. At first, the disease activity in the study group was relatively low. The prevalence of new cavities was 35%, ranging from 15% in very low risk category to 55% in the very high risk category. A higher disease would likely have increased the positive predictive values in a substantial way. Secondly, the vast majority of the patients was recalled by their regular team during the course of the study and provided with various forms of preventive care. Although the Cariogram risk category was not informed, the patients were continuously made aware of their risk category according to the public dental clinics guidelines [15], and, at best, this should have influenced the treatment decisions of the dental personnel. Evidently, a successful preventive care could impair the predictive abilities of any caries risk program. Any restorative treatment decision was taken by the regular dentist and no specific recommendations or guidelines were issued for this study. Therefore, we used total caries index (DFT/DFS) rather than the D-component to reflect the three year caries increment. The third issue that could have influenced the outcome was the 24% dropout rate. Obviously, the dropouts displayed a higher burden of disease and caries risk factors and it is well known that those with active caries are most likely to develop more caries [6] and furthermore, the most caries susceptible patients were underrepresented among those that consented to the baseline examination, indicating a selection bias [15, 17]. For example, if one assumes that 80% of the dropouts would have developed new cavities over the study period, the sensitivity and positive predictive values would have increased by approximately 10%. Thus, it is important to keep in mind that the figures obtained in the present study were based on this particular population, under given circumstances, and cannot readily be generalized.

## Conclusions

In conclusion, within the limitations of the present study, the computer-based Cariogram did not perform better than a caries risk assessment scheme based on past caries experience and caries progression, over a 3-year period in young adults.

## References

[1] Twetman S, Fontana M (2009). Patient caries risk assessment. Monogr Oral Sci.

[2] Twetman S, Fontana M, Featherstone JD (2013). Caries risk assessment – can we achieve consensus?. Community Dent Oral Epidemiol.

[3] Hallett KB (2013). The application of caries risk assessment in minimum intervention dentistry. Aust Dent J.

[4] Riley JL, Qvist V, Fellows JL, Rindal DB, Richman JS, Gilbert GH (2010). Dentists’ use of caries risk assessment in children: findings from the Dental Practice-Based Research Network. Gen Dent.

[5] Riley JL, Gordan VV, Ajmo CT, Bockman H, Jackson MB, Gilbert GH (2011). Dentists’ use of caries risk assessment and individualized caries prevention for their adult patients: findings from The Dental Practice-Based Research Network. Community Dent Oral Epidemiol.

[6] Mejare I, Axelsson S, Dahlén G, Espelid I, Norlund A, Tranaeus S (2014). Caries risk assessment. A systematic review. Acta Odontol Scand.

[7] Bratthall D, Hänsel PG (2005). Cariogram–a multifactorial risk assessment model for a multifactorial disease. Community Dent Oral Epidemiol.

[8] Holgerson PL, Twetman S, Stecksèn-Blicks C (2009). Validation of an age-modified caries risk assessment program (Cariogram) in preschool children. Acta Odontol Scand.

[9] Hänsel Petersson G, Twetman S, Bratthall D (2002). Evaluation of a computer program for caries risk assessment in schoolchildren. Caries Res.

[10] Campus G, Cagetti MG, Sale S, Carta G, Lingström P (2012). Cariogram validity in schoolchildren: a two-year follow-up study. Caries Res.

[11] Petersson GH, Isberg PE, Twetman S (2010). Caries risk assessment in school children using a reduced Cariogram model without saliva tests. BMC Oral Health.

[12] Zukanović A (2013). Caries risk assessment models in caries prediction. Acta Med Acad.

[13] Alian AY, McNally ME, Fure S, Birkhed D (2006). Assessment of caries risk in elderly patients using the Cariogram model. J Can Dent Assoc.

[14] Celik EU, Gokay N, Ates M (2012). Efficiency of caries risk assessment in young adults using Cariogram. Eur J Dent.

[15] Hänsel Petersson G, Ericson E, Isberg PE, Twetman S (2013). Caries risk assessment in young adults using Public Dental Service guidelines and the Cariogram-a comparative study. Acta Odontol Scand.

[16] WHO (1987). Oral health surveys: basic methods.

[17] Hänsel Petersson G, Ericson E, Isberg PE, Twetman S (2013). Caries risk assessment in young adults: a 3-year validation of clinical guidelines used in Public Dental Service. Acta Odontol Scand.

[18] Tellez M, Gomez J, Pretty I, Ellwood R, Ismail A (2012). Evidence on existing caries risk assessment systems: are they predictive of future caries?. Community Dent Oral Epidemiol.

[19] Gao X, Di Wu I, Lo EC, Chu CH, Hsu CY, Wong MC (2013). Validity of caries risk assessment programmes in preschool children. J Dent.

[CR20] The pre-publication history for this paper can be accessed here:http://www.biomedcentral.com/1472-6831/15/17/prepub

